# Assessment of Self-Medication Behaviour in Response to Dental Pain in Two Populations, France

**DOI:** 10.1016/j.identj.2026.109418

**Published:** 2026-02-04

**Authors:** Louise Le Texier, Chantal Savanovitch, Emmanuel Nicolas, Pierre-Yves Cousson

**Affiliations:** a Université Clermont Auvergne, CROC, Clermont-Ferrand, France; b Faculty of Pharmacy, University of Clermont Auvergne, Clermont-Ferrand, France; c Research Unit ACCePPT, Clermont-Ferrand, France; d CHU Clermont-Ferrand, Service d’Odontologie, Clermont-Ferrand, France

**Keywords:** Oral health, Self-medication, Behaviours, Prevalence, Dental pain

## Abstract

**Introduction and aims:**

Self-medication appears to be a common practice for dental pain. However, in France, its prevalence and patterns in dentistry have never been studied. The primary objective was to assess the prevalence and self-medication behaviours in two at-risk populations: patients consulting for acute pulpal or periapical pain and patients with dental anxiety requiring treatment under general anaesthesia. The secondary objective was to examine the influence of socio-behavioural factors on these practices.

**Method:**

Between April 2021 and May 2023, the behaviours of two at-risk population regarding self-medication were analysed in a cross-sectional observational study. The first population regrouped patients referred to an endodontic postemergency care unit after visiting the emergency service of a dental hospital. These patients were referred due to acute pulpal or periapical pain (Endodontic Group). The second population regrouped patients referred to a special care unit for dental treatment under general anaesthesia due to dental anxiety (Anxiety Group). Self-medication behaviours of the two at-risk populations were analysed with 5 self-administered questionnaires (self-medication, EPICES, IDAF-4C, Pain Catastrophizing Scale, Socio-demographic data). Comparisons between the two population were done using Pearson’s chi-square and Student’s *t* tests.

**Results:**

During the study period, 43 patients were included in the endodontic group and 66 in the anxiety group. Socio-demographic and behavioural data differed between the two groups. However, self-medication prevalence was similar (51.2% in the Endodontic Group *vs* 45.5% in the Anxiety Group), as were self-medication behaviours (types and number of substances used, methods of acquisition, knowledge). No socio-demographic or behavioural factors explained these attitudes.

**Conclusion:**

Self-medication in dentistry is often overlooked or poorly managed. Preventive measures and patient education on the proper use of medication are essential.

**Clinical relevance:**

Standardized cooperation protocols should be developed involving dentists and community pharmacists to optimize the management of patients suffering from dental pain.

## Introduction

According to the World Health Organization, self-medication is defined as individual uses medicine on their own initiative to treat a self-identified disorder or symptom, without consulting a doctor.^1^ A distinction is made between optional medical prescription drugs, available without a prescription, and mandatory prescription drugs. Self-medication may involve the use of medications that are available without prescription and/or seeking medical advice. It may also include the use of leftover medicines from previous prescriptions that remain accessible in the home medicine cabinet. Uncontrolled self-medication includes excessive drug use (polypharmacy), inappropriate antimicrobial use, often in inadequate doses for nonmicrobial infections – and frequent misuse of prescription-only drugs. These behaviours can delay proper treatment, increase the risk of drug interactions, and lead to overdosing, all of which pose significant health risks.^2^ Self-medication is widespread across countries^3^, ^4^, ^5^ and populations.^6^^,^^7^ Its prevalence in Europe and North America ranges from 8% to 13%.^8^ In 2017, in France, the IPSOS statistics institute estimated that eight out of 10 people had engaged in self-medication.^9^ During COVID-19 pandemic, self-medication behaviours were modified. Two systematic reviews^10^^,^^11^ showed that prevalence of self-medication varied greatly between countries but also between populations, ranging from 7% to 88%. Dental problems were no exception to this trend. Oral diseases were treated at home with analgesics, antibiotics, or traditional remedies. An Iran cross-sectional study showed that self-medication with antibiotics for dental pain was more prevalent after the pandemic.^12^

In dentistry, acute pain is most often caused by pulpal or periapical inflammatory (pulpitis) or infectious (acute apical abscess).^8^^,^^13^ Pulpitis is an inflammation of the pulp in response to irritants of microbial, chemical or physical origin.^14^ Acute apical abscess is an invasion of the pulp by bacteria and an extension of the infectious process to the entire canal system and ligament.^15^ Patients typically describe their pain as moderate to severe.^13^^,^^16^, ^17^, ^18^ These acute pains are generally managed in the emergency settings.^8^^,^^13^ The only effective treatment for endodontic disease requires a dentist’s technical intervention to remove the source of inflammation or infection. However, studies indicate that patients experiencing dental pain often self-medicate before seeking professional care.^19^^,^^20^ This behaviour appears to be influenced by socio-behavioural factors such as social status, perception of illness, and/or anxiety or fear of dental care.^21^^,^^22^ Other studies have also linked self-medication to difficulties in accessing emergency dental care,^22^ lack of time^23^ and financial constraints.^24^

A recent systematic review identified only 37 studies on self-medication for dental pain conducted between 2011 and 2023, primarily in developing countries.^20^ The prevalence of self-medication for dental pain ranged from 21.8% in Malaysia^25^ to 89% in Madhya Pradesh region, India.^23^ Acetaminophen and ibuprofen, both available without prescription, are the most commonly used pain relievers.^26^^,^^27^ However, they are only effective in 46% to 62% of cases, and provide short-term relief.^13^ Numerous case reports have highlighted unintentional acetaminophen overdose when used for dental pain.^28^, ^29^, ^30^ Patients with dental pain are 12.8 times more likely to overdose on acetaminophen than patients with other types of pain,^31^ and 41% of hospital admissions for unintentional acetaminophen overdose were related to dental pain.^32^

In France, no studies have investigated self-medication behaviour for dental pain.

This study aimed at investigating self-medication practices in two at-risk populations: patients seeking care for acute pulpal or periapical dental pain in a dental hospital, and patients with dental anxiety consulting for care under general anaesthesia. The primary objective was to determine the prevalence and patterns of self-medication. The secondary goal was to determine whether socio-behavioural factors influence these practices.

## Material and method

Between April 2021 and May 2023, the behaviours of two at-risk populations regarding self-medication were analysed in a cross-sectional observational study. The first population regrouped patient referred to an endodontic postemergency care unit of a dental hospital due to acute pulpal or periapical pain (Endodontic Group: EndoG). The second population regrouped patient referred to a special care unit for dental treatment under general anaesthesia due to dental anxiety (Anxiety Group: AnxG). The inclusion criteria were patients over 18 years old, fluent in French, and without guardianship. Ethical approval was obtained from the ethics committee under the number 2021-A00417-34, in June 2021, and nonopposition to participate were given. Patients were asked to complete 5 questionnaires.

1. The self-medication questionnaire assesses prevalence of self-medication regardless of context with the question: ‘Do you self-medicate?’, and self-medication behaviours in two contexts: Pain setting (use of self-medication before or during the preconsultation period in an emergency setting) and in General setting (focused on self-medication in the management of dental pain, outside of emergency contexts). Self-medication behaviours were evaluated in terms of prevalence, medications consumed, dose, and frequency. This questionnaire also assessed knowledge of drugs ‘maximum doses and associated risks’;
2. Dental Anxiety and Phobia Questionnaire (IDAF-4C)^33^;
3. the EPICES index (Evaluation de la Précarité et des Inégalités de santé dans les Centres d’Examen de Santé. Evaluation of Precariousness and Health Inequalities in Health Examination Centers)^34^^,^^35^;
4. Pain Catastrophizing Scale (PCS)^36^;
5. a socio-demographic questionnaire.

Data from the self-medication questionnaire served as the dependent variable, while data from the other questionnaires were the explanatory factors for self-medication.

The required sample size was calculated using data from a preliminary pilot study assessing the prevalence of self-medication. This pilot study showed that 20 out of 30 patients with acute pulpal pain (66.7%) and 27 out of 30 anxious patients (90%) reported self-medication. Based on these prevalence estimates in the two groups, the sample size calculation indicated that at least 40 participants per group were required (EpiR package, *α* = 0.05; 1 – *β* = 0.8; two-sided test).

Data were analysed using SPSS Statistic v29 (IBM). A descriptive analysis of the self-medication questionnaire data was performed for each group. The prevalence of self-medication, number and type of medication, and strategy for obtaining medication were determined separately for each group in the general and pain settings. Socio-demographic and behavioural characteristics were also examined. Results of self-medication in the general setting were compared using a Pearson Chi-square test (*P* < .05) and a Student’s *t* test (*α* = 0.05). A statistical comparison of self-medication in pain setting was not performed due to the small number of respondents in that group.

Pearson correlation analysis followed by linear regression was conducted to determine the explanatory factors for self-medication in each group.

## Results

### Population description

A total of 109 participants were included, with 43 in the Endodontic Group (EndoG) and 66 in the Anxiety Group (AnxG). Socio-demographic and behavioural characteristics are presented in Table 1. EndoG and AnxG were different in terms of age, gender, precariousness, pain catastrophizing, dental fear, and anxiety scores. The ‘typical patient’ in EndoG was a man in his thirties, blue-collar, with no more than 2 years postgraduate education and in a nonprecarious situation. The ‘typical patient’ in AnxG was a 36-year-old woman, blue-collar, with a maximum of 2 years’ higher education, and living in precarious conditions. Furthermore, when asked ‘Do you self-medicate?’, patients in AnxG responded significantly more often ‘yes’ than patients in EndoG (86.4% vs 69.8%, respectively [*P* = .035]).

**Table 1:** Distribution of socio-demographic and behavioural characteristics for endodontic and anxiety groups.

	Endodontic group *N* = 43 (%) Mean ± SD	Anxiety group *N* = 66 (%) Mean ± SD	Significance	Test used
Gender				
Female	16 (37.2%)	47 (71.2%)	*P* < .001*	Pearson’s Chi²
Male	27 (62.8%)	19 (28.8%)		
Age (y)	30 ± 10.2	35.7 ± 11.2	*P* = .004*	Student’s *t* test
Home department				
Allier and Puy de Dome	41 (95.3%)	50 (75.8%)	*P* = .061	Pearson’s Chi²
Other	2 (4.7%)	10 (15.1%)		
NA	0	6 (9.1%)		
Place de residence				
Town	34 (79.1%)	35 (53.1%)	*P* = .066	Pearson’s Chi²
Suburb	3 (7%)	3 (4.5%)		
Country	6 (13.9%)	20 (30.3%)		
NA	0	8 (12.1%)		
Degree				
None	7 (16.3%)	8 (12.1%)	*P* = .269	Pearson’s Chi²
≤2 y postgraduation	25 (58.1%)	37 (56.1%)		
≥3 y postgraduation	9 (20.9%)	6 (9.1%)		
NA	2 (4.7%)	15 (22.7%)		
Profession				
White-collar workers	2 (4.7%)	2 (3%)	*P* = .872	Pearson’s Chi²
Intermediate professions	5 (11.6%)	5 (7.6%)		
Farmer	0	5 (7.6%)		
Blue-collar workers	20 (46.5%)	31 (46.9%)		
Student	6 (14%)	5 (7.6%)		
Unemployed	5 (11.6%)	5 (7.6%)		
Retired	1 (2.3%)	1 (1.5%)		
NA	4 (9.3%)	12 (18.2%)		
Score EPICES – Mean score	29.9 ± 18	36.7 ± 18.7	*P* = .032*	Student’s *t* test
PCS – Mean score	22.3 ± 12.4	32.9 ± 13.3	*P* < .001*	Student’s *t* test
IDAF 1				
Mean score	19.1 ± 9.7	36.1 ± 6.1	*P* < .001*	Student’s *t* test
No anxiety	6 (14%)	0		
Significant anxiety	1 (2.3%)	25 (37.9%)		
IDAF 2				
Mean score	9.1 ± 1.2	6.5 ± 1.3	*P* < .001*	Student’s *t* test
Dental phobia	0	18 (27.3%)		
No dental phobia	24 (55.8%)	3 (4.5%)		
NA	0	1 (1.5%)		
Do you self-medicate?				
Yes	30 (69.8%)	57 (86.4%)	*P* = .035*	Pearson’s Chi²
No	13 (30.2%)	9 (13.6%)		

EPICES, Evaluation de la Précarité et des Inégalités de santé dans les Centres d’Examen de Santé; IDAF, Index of Dental Anxiety and Fear; NA, no answer; PCS, Pain Catastrophizing Scale.

⁎ Significant.

### Self-medication in general setting

The number of drugs used in self-medication was comparable in both groups, respectively 1.8 for AnxG *vs* 1.5 drugs for EndoG (*P* = .098). Two drugs were primarily used: acetaminophen and ibuprofen. AnxG patients consumed significantly more acetaminophen associated with codeine than EndoG patients (24.2% vs 9.3%, *P* = .049). Other substances used included tramadol, ketoprofen, and antibiotics (Table 2).

**Table 2:** Distribution of type of medication and frequency of consumption for endodontic and anxiety groups in general setting.

Medications	Endodontic group *N* = 43 (%)	Anxiety group *N* = 66 (%)	Significance Pearson Chi-square test
Acetaminophen	34 (79.1%)	45 (68.2%)	*P* = .214
Acetaminophen + codeine	4 (9.3%)	16 (24.2%)	*P* = .049*
Tramadol	5 (11.6)	7 (12.2%)	*P* = .938
Ibuprofen	11 (25.6%)	22 (33.3%)	*P* = .389
Ketoprofen	1 (2.3%)	2 (3%)	*P* = .826
Antibiotics	1 (2.3%)	11 (16.7%)	*P* = .019*
Others	0	6 (9.1%)	*P* = .042*

⁎ Significant.

Both groups obtained their medication through similar means: ‘self-medication without advice’, ‘pharmacist’ advice’, and ‘doctor practitioner’ (Table 3).

**Table 3:** Distribution of strategy of obtaining medication consumed for endodontic and anxiety groups in general setting.

Method	Endodontic group *N* = 43 (%)	Anxiety group *N* = 66 (%)	Significance Pearson Chi-square test
Pharmacist’ advice	5 (11.6%)	10 (15.2%)	*P* = .106
Calling an emergency service	0	6 (9.1%)	
Self-medication without advice	22 (51.2%)	30 (45.5%)	
Pharmacist’ advice + Self-medication	3 (7%)	1 (1.5%)	
Calling an emergency service + Self-medication	0	2 (3%)	
Doctor practitioner + Self-medication	0	2 (3%)	
Pharmacist’ advice + calling an emergency service + Self-medication	0	1 (1.5%)	
Doctor practitioner	1 (2.3%)	7 (10.6%)	
NA	6 (14%)	2 (3%)	
NR	6 (14%)	5 (7.6%)	
Total	43 (100%)	66 (100%)	

NA, not answer; NR, not requested.

No socio-demographic or behavioural factors were found to explain self-medication in the context of oral health care.

### Self-medication in pain setting – Before visiting

In pain setting, 34.9% of EndoG patients experienced pain for less than 48 hours, 39.5% for a week, 23.3% for more than 2 weeks prior visiting. The mean number of consumed drugs to relieve their pain was 1.5 medications (min: 0 to max: 5). Acetaminophen was the most used drug (79.1%), followed by ibuprofen (23.3%) (Table 4). Six patients had consumed more than 4000 mg/d of acetaminophen, and two participants had consumed 1600 mg of ibuprofen. Most of them (51.2%) self-medicated without any medical advice (Table 5).

**Table 4:** Distribution of type of medication and frequency of consumption for endodontic and anxiety groups in pain setting, before the appointment.

Medications	Endodontic group *N* = 43 (%)	Anxiety group *N* = 12 (%)
Acetaminophen	34 (79.1%)	7 (58.3%)
Acetaminophen + codeine	5 (2.4%)	5 (41.7%)
Tramadol	7 (16.3%)	3 (25%)
Ibuprofen	10 (23.3%)	5 (41.7%)
Ketoprofen	0	1 (8.3%)
Antibiotics	7 (16.3%)	4 (33.3%)
Others	3 (6.9%)	0

**Table 5:** Distribution of strategy of obtaining medication consumed for endodontic and anxiety groups in pain setting, before the appointment.

Method	Endodontic group *N* = 43 (%)	Anxiety group *N* = 12 (%)
Pharmacist’ advice	8 (18.6%)	3 (25%)
Calling an emergency service	4 (9.3%)	2 (16.7%)
Self-medication without advice	22 (51.2%)	5 (41.7%)
Pharmacist’ advice + Self-medication	3 (6.9%)	0
Calling an emergency service + Self-medication	2 (4.7%)	0
Doctor practitioner	0	1 (8.3%)
NA	4 (9.3%)	1 (8.3%)
Total	43 (100%)	12 (100%)

NA, not answer; NR, not request.

Among the 66 AnxG patients, 12 declared to have pain for more than 2 weeks, of which nine presented chronic pain (more than 3 months). The mean number of consumed drugs to relieve their pain was 2.1 drugs (min: 1 to max: 4). Acetaminophen was the most used drug (58.3%), followed by ibuprofen (41.7%) and codeine acetaminophen (41.7%) (Table 4). Two participants had overconsumed acetaminophen. Most of them are self-medicated without medical advice (41.7%) (Table 5).

### Knowledge

Similar results were observed in both groups regarding knowledge of the maximum daily dose allowed for medications and the risks associated with drug misuse. For acetaminophen, 43.2% of EndoG participants and 36.4% of AnxG participants knew the maximum daily dose. For ibuprofen, 50% of the EndoG subjects and 41.7% of the AnxG subjects knew the maximum daily dose (*P* = .479).

## Discussion

This study aimed at determining the prevalence and patterns of self-medication in two at-risk populations, one visiting an emergency unit for pain of pulpal or periapical origin (EndoG) and the other suffering from anxiety and/or phobia about dental care (AnxG).

The assessment of self-medication habits revealed a statistically similar prevalence of self-medication in both groups (around 57%), in line with the literature.^27^ The most frequently used substance were acetaminophen and ibuprofen, in accordance with previous studies.^26^^,^^27^ Since these medications are available without prescription, they are easily accessible and inexpensive (approximately 2€). Moreover, these are even widely advertised as effective for dental pain relief. In emergency services, advice is often given to take 1 g of acetaminophen in case of dental pain. This various information available to the public hint that there is no danger in taking acetaminophen. Furthermore, patients’ knowledge of maximum daily doses for acetaminophen and ibuprofen was limited, but comparable between the two groups. Additionally, some patients were also unaware that the same active substance could be available under different brand names. All these factors increase the risk for overdose. The number of used drugs (1.8 EndoG vs 1.5 AnxG) was statistically comparable between the two groups, and in line with previous studies.^24^^,^^26^ The primary source of self-medicated drugs was the patients’ own medicine cabinet. Although the origin of these medications was not specifically investigated, it is likely that they were remnants of previous prescriptions. Some patients also reported using other substances, such as opioids or antibiotics. The risks associated with taking these drugs without professional guidance are significant. In France, a distinction is made between inaccessible (behind-the-counter) and over-the-counter medicines with optional prescription medicines including nonsteroidal anti‑inflammatory drug and acetaminophen. In order to optimize the proper use of these commonly used drugs, the Agence Nationale de la Sécurité du Médicament (ANSM, National Agency for the Safety of Medicines and Health Products) has decided, from 15 January 2020, that these drugs may no longer be presented as over-the-counter in all pharmacies, to promote community pharmacist advice.^37^ Thus, the pharmacist has a responsibility to give an advice when providing medicines that do not require a medical prescription (According to Article R. 4235-48 of the Public Health Code^38^). Modifications in prescribing and dispensing medications have also been decided so that codeine drugs could no longer be sold over the counter based on the decree of 12 July 2017. Because of the occurrence of overdose, the French authority emphasized on the necessity of medical prescription related to the intake of the above drugs in order to avoid the misuse by adolescents.^39^ Since 2025,^40^ community pharmacists have been able to offer support interviews to patients on opioid analgesics (level II), such as tramadol and codeine. The objective is to identify or prevent misuse or dependence on these drugs. The misuse of antibiotics (unjustified use or inappropriate daily dosage) contributes to bacterial resistance.

When pain was present (EndoG group), the prevalence of self-medication was 69.2%, comparable to previous studies in Nepal (63%)^41^ and the United Arab Emirates (70%).^2^ However, lower prevalences were reported on studies carried out in Karnataka region, India (30%)^42^ and Belgium (40.8%).^26^ In this study, patients used an average of 1.5 different medications, mainly acetaminophen and ibuprofen, which was also found in previous studies.^26^^,^^27^^,^^43^ In 62.8% of cases, patients sought consultation after experiencing pain for more than a week. This prolonged delay raises concerns regarding the time required for managing dental conditions and the potential risk of analgesic misuse (eg, prolonged use or overconsumption). Current health recommendations^44^^,^^45^ advise consulting a healthcare professional if fever persists for more than 3 days or if pain persists for more than 5 days despite taking analgesic. Studies have also suggested that these medications have limited efficacy in managing endodontic pain.^46^ In 2020, the COVID-19 pandemic has modified self-medication behaviour. Indeed, lockdown measures overwhelmed healthcare facilities, induced fear of infection during consultations, and difficulty accessing a healthcare professional quickly led a large part of the population to manage their symptoms on their own. Two systematic reviews showed that, overall, prevalence of self-medication varied from 7% to 88%. Several studies have examined self-medication in dentistry during COVID-19 pandemic. Although one study showed that prevalence of self-medication for dental pain was similar before and after COVID-19,^47^ others have highlighted an increase in this practice. In Pakistan, the prevalence of self-medication increased from 71.4% to 86.2%.^48^ In Iran, the use of antibiotics without medical advice doubled.^12^ In the present study, conducted after the various lockdowns, self-medication in the two populations studied was high. However, as no such study had been conducted in France prior to 2020, it was not possible to compare self-medication behaviours before and after the pandemic.

Further studies, particularly qualitative studies, are needed to explore the reasons behind delayed consultation. The findings could help improve emergency care pathways and the management of dental pain. To gain better understanding of self-medication behaviours of these two at-risk populations, a qualitative study using semistructured interviews should be carried out to identify potential explanatory factors or motivations. This would help define targeted interventions to promote safer self-medication practices.

This study allowed for the characterization of the two populations analysed. Although their self-medication behaviours were comparable, they differed in terms of age, gender, precariousness, anxiety about dental care, and pain catastrophizing. The socio-demographic differences observed were linked to their reason for consultation. EndoG Patients consulted urgently for pain management and were scheduled for an appointment on the same day or the following day. The ‘typical patient’ for this group was a man in his thirties, blue- or white-collar, with no more than 2 years postgraduate education and in a nonprecarious situation. Similar patient profiles have been reported in studies on self-medication in dentistry by AlQahtani^2^ and Azodo.^49^ Geographically, EndoG patients were living closer (95% Puy-de Dôme or Allier) to the emergency unit. In other furthest departments, patients tended to find a dental surgeon or general practitioner closer to home, avoiding the long journey to come to the dental emergency department. Conversely, AnxG patients scheduled their appointments in advance for dental treatment under general anaesthesia due to their severe dental anxiety, as confirmed by high IDAF1 and 2 scores. The ‘typical patient’ in this group was a 38-year-old woman, blue- or white-collar, with a maximum of 2 years’ higher education and living in precarious conditions. These data were in line with Armfield’s study of dental care anxiety in Australia.^50^ AnxG patients sought specialized care that is not widely available, which explains their diverse geographical origins – 15.2% came from outside the Puy-de-Dôme and Allier departments. Additionally, 62.1% of AnxG patients were in precarious situations. A qualitative study, conducted in 2018 on patients who had undergone general anaesthesia for dental treatment, found that many were in precarious situations and that general anaesthesia provided them with the most suitable access to care.^51^

The secondary objective of this study was to identify whether any socio-behavioural factors might explain self-medication behaviour. Unlike previous studies that have identified age,^52^ gender,^42^ level of education^24^ or socio-professional category^42^ as explanatory factors for self-medication, none of the socio-demographic variables analysed in this study were found to be significant. Similarly, no behavioural factors (PCS, IDAF) were identified as influencing self-medication behaviour.

The PCS, which assesses the mechanisms by which catastrophizing influences the patient’s experience of pain, was used for the first time in a dental context. Studies conducted by Sullivan^53^, ^54^, ^55^ have shown that a PCS score >30 indicated a clinically significant level of pain catastrophization and may serve as a predictor of chronic pain development. In this study, the mean PCS score for AnxG was 32.9, and 75% of patients reported chronic pain.

One limitation was the selection bias used to create the EndoG group, as only patients suffering from endodontic pain and managed in the endodontic postemergency unit were included. It is possible that some patients with endodontic pain were not referred to this unit due to prior drug overdose or scheduling constraints. Futures studies should consider including patients upon their initial arrival at the hospital dental emergency department to obtain a more comprehensive sample. Another limitation concerned the self-medication questionnaire: the questions were not adapted to the two populations and focused mainly on methods and knowledge of self-medication. Reasons for self-medication, which could explain these behaviours, were not sought. These limitations were mainly related to the design of the questionnaire and the difficulty in interpreting questions such as ‘What do you use in case of self-medication, regardless of context?’ and ‘What do you use when you are in pain in a general setting’. A new questionnaire should be developed, taking these limitations into account, to better assess the prevalence of self-medication and its causes. Moreover, developing a validated self-medication questionnaire applicable across various medical specialties would facilitate large-scale studies on self-medication behaviours and enable cross-specialty comparisons.

## Conclusion

This study, the first in France, aimed at describing the prevalence and self-medication behaviour in two at-risk populations. The self-medication habits of the two populations were similar. The lack of knowledge regarding medications underscores the need for drug education supported by practical measures. Further qualitative studies are needed to better understand the underlying motivations for self-medication. These studies could contribute to improving protocols for access to emergency care, preventing high-risk practices, and promoting ‘responsible’ self-medication. Standardized cooperation protocols should be developed involving dentists and community pharmacists to optimize the management of patients suffering from dental pain.

## Author contributions

Louise Le Texier: Conceptualization, methodology, investigation, writing original draft preparation; Emmanuel Nicolas: Formal analysis, writing – reviewing; Chantal Savanovitch: Writing – reviewing; Pierre-Yves Cousson: Conceptualization, methodology, investigation, writing-reviewing, supervision.

## Ethics statement and consent to participate

This study was conducted in full accordance with the Declaration of Helsinki. Ethical approval was obtained from the local ethical committee (2021-A00417-34, date 11/06/2021). Patients received information about the study and were given opportunity to oppose participation in this study.

## Funding

This research did not receive any specific grant from funding agencies in the public, commercial, or not-for-profit sectors.

## Data availability

Data will be available upon request to the authors.

## Conflict of interest

The authors declare that they have no known competing financial interests or personal relationships that could have appeared to influence the work reported in this article.
